# Anti-Inflammatory and Prebiotic Potential of Ethanol Extracts and Mucilage Polysaccharides from Korean Yams (*Dioscorea polystachya* and *Dioscorea bulbifera*)

**DOI:** 10.3390/foods14020173

**Published:** 2025-01-08

**Authors:** So-Yoon Park, Van-Long Truong, Su-Gyeong Jeon, So-Young Choe, Razanamanana H. G. Rarison, Byoung-Hoon Yoon, Ji-Won Park, Hye-Jeong Jeong, Woo-Sik Jeong

**Affiliations:** 1 School of Food Science & Biotechnology, College of Agriculture and Life Sciences, Kyungpook National University, Daegu 41566, Republic of Korea; thdbs1958@naver.com (S.-Y.P.); truonglongpro@gmail.com (V.-L.T.); rhanitranirina@gmail.com (R.H.G.R.); byounghoon97@naver.com (B.-H.Y.); yozi6789@naver.com (J.-W.P.); rkawktnsdl12@naver.com (H.-J.J.); 2 Food and Bio-Industry Research Institute, School of Food Science & Biotechnology, College of Agriculture and Life Sciences, Kyungpook National University, Daegu 41566, Republic of Korea; 3 Institute for Bioresources Research, Gyeongsangbuk-do Agricultural Research and Extension Services, Andong 36614, Republic of Korea; grapecorn@korea.kr (S.-G.J.); foodgirl02@korea.kr (S.-Y.C.)

**Keywords:** *Dioscorea polystachya*, *Dioscorea bulbifera*, Korean yam, polysaccharides, anti-inflammation, immune, prebiotics

## Abstract

Korean yams are abundant in bioactive compounds with significant health-promoting properties. This study evaluated the anti-inflammatory potential of ethanol and water extracts from *Dioscorea polystachya* and *Dioscorea bulbifera* in RAW 264.7 macrophage cells. Among the extracts, the 95% ethanol extract exhibited the most potent inhibition of reactive oxygen species (ROS) and nitric oxide (NO) production, warranting further exploration of its mechanisms of action. Further analysis revealed that the ethanol extract modulated key inflammatory signaling pathways, including MAPK and NF-κB, contributing to its anti-inflammatory activity. Additionally, mucilage polysaccharides, a key bioactive component of Korean yams, were extracted and characterized for their structural and functional properties. These polysaccharides demonstrated immune-enhancing effects by reducing ROS and NO production while increasing phagocytic activity in the RAW 264.7 cells. Their prebiotic potential was also assessed through microbial growth assays, which showed an enhanced proliferation of beneficial bacteria such as *Lactobacillus* and *Bifidobacterium*. Furthermore, the adhesion assays using Caco-2 intestinal epithelial cells revealed that these polysaccharides promoted probiotic adhesion while inhibiting the adhesion of pathogenic bacteria. These findings highlight the bioactive potential of ethanol extracts and mucilage polysaccharides from Korean yams, emphasizing their promising applications as anti-inflammatory, immune-modulating, and prebiotic agents for functional food and nutraceutical development.

## 1. Introduction

Korean yams, particularly *Dioscorea polystachya* and *Dioscorea bulbifera*, have been extensively utilized in traditional medicine for their notable nutritional and therapeutic properties [1]. These tubers, commonly consumed in various traditional dishes and remedies, hold a prominent place in dietary and medicinal practices across Asia [2]. They are abundant in physiologically active constituents, including mucilage polysaccharides, flavonoids, saponins, and phenolic acids, which are associated with a wide range of health benefits, ranging from metabolic regulation to immune enhancement [3]. While their traditional applications are well documented, contemporary research has increasingly focused on isolating these bioactive compounds and elucidating their underlying mechanisms, thereby facilitating their incorporation into modern therapeutic frameworks and functional food systems [4].

Among the diverse physiological effects attributed to these compounds, their potential to mitigate inflammation and oxidative stress has drawn considerable attention from the scientific community [5]. Inflammation and oxidative stress are critical drivers of numerous chronic diseases, including cardiovascular disorders, diabetes, and inflammatory bowel disease, which collectively account for a significant amount of the global disease burden [6,7,8]. Chronic inflammation, often stemming from dysregulated immune responses, results in excessive production of reactive oxygen species (ROS) and nitric oxide (NO), which are strongly implicated in the progression of metabolic syndromes and the associated complications [9]. Investigating the interplay between these bioactive molecules and pathological pathways offers valuable insights into their therapeutic potential, with promising implications for preventive and therapeutic medicine [10].

Recent studies have reported the anti-inflammatory properties of Korean yams, particularly focusing on ethanol and aqueous extracts. For instance, Kim and Kim demonstrated the anti-inflammatory effects of ethanol extracts from *D. bulbifera*, showing a significant inhibition of inflammatory markers in vitro [11]. Similarly, Mbiantcha et al. reported the analgesic and anti-inflammatory properties of extracts from the bulbils of *D. bulbifera* var. sativa in murine models, underscoring their therapeutic potential [12]. These findings complement the existing knowledge by providing further evidence of the therapeutic potential of the *Dioscorea* species in managing inflammation.

Mucilage polysaccharides, one of the key bioactive components in Korean yams, have emerged as particularly promising candidates for modulating these inflammatory and oxidative processes [13,14,15]. These naturally derived biopolymers exert immunomodulatory effects by activating macrophages, enhancing phagocytosis, and regulating cytokine production, which collectively contribute to a balanced and robust immune response [16,17]. Moreover, their prebiotic properties are well established, as they support the proliferation of beneficial gut microbiota, such as Lactobacillus and Bifidobacterium, while simultaneously suppressing the growth of pathogenic species like *Escherichia coli* [18]. This dual functionality not only contributes to improved gut health but also strengthens immune responses via the gut–immune axis, underscoring their potential as therapeutic agents in combating inflammation, oxidative stress, and related chronic conditions [19].

This study investigates the anti-inflammatory effects of ethanol and water extracts derived from *D. polystachya* and *D. bulbifera*. These extracts were rigorously evaluated in RAW 264.7 macrophage cells to determine their ability to inhibit ROS and NO production and to regulate inflammatory signaling pathways, with a particular focus on the MAPK (mitogen-activated protein kinase) and NF-κB (nuclear factor-kappa B) pathways, which are central mediators of inflammation. In addition, mucilage polysaccharides were extracted and characterized in terms of their structural and functional properties to better understand their bioactivities. Their immune-modulating potential was assessed by measuring their effects on ROS and NO production, as well as macrophage phagocytic activity, while their prebiotic effects were evaluated through microbial growth assays and adhesion tests using Caco-2 intestinal epithelial cells. These assays demonstrated the ability of mucilage polysaccharides to promote the adhesion of beneficial bacteria while inhibiting the colonization of harmful pathogens, further emphasizing their role in maintaining gut health and systemic immune balance.

By integrating anti-inflammatory, immune-modulating, and prebiotic properties, the bioactive compounds in Korean yams demonstrate a multifunctional approach to health promotion. The findings of this comprehensive study not only highlight the therapeutic potential of ethanol extracts and mucilage polysaccharides from *D. polystachya* and *D. bulbifera* but also underscore their broader applicability in functional foods and nutraceuticals. These compounds provide natural, plant-based solutions for addressing chronic inflammation, supporting immune resilience, and promoting a healthy gut microbiome [20,21]. As the global demand for sustainable, plant-derived health products continues to grow, the insights provided by this research offer valuable opportunities for the development of innovative health-promoting interventions that bridge the gap between traditional medicine and modern science.

## 2. Materials and Methods

### 2.1. Materials

Lipopolysaccharide (LPS, *Escherichia coli* O127:B18), Dimethyl sulfoxide (DMSO), formalin, 2′,7′-dichlorofluorescein diacetate (DCFH-DA), Anthrone, 3-(4,5-dimethylthiazol-2-yl)-2,5-diphenyltetrazolium bromide (MTT), Alginic acid sodium salt, Trifluoroacetic acid (TFA), and sulforaphane (SFN) were purchased from Sigma-Adrich (St. Louis, MO, USA). Sulfuric acid and ethanol were purchased from Duksan Pure Chemicals Co. (Ansan-si, Gyeonggi-do, Republic of Korea). Neutral red, carbazole, and glucuronic acid were obtained from Daejung Co. (Gyeonggi-do, Republic of Korea). Anti-inducible nitric oxide synthase (iNOS), cyclooxygenase-2 (COX-2), tumor necrosis factor-α (TNF-α), inhibitory κB kinase αβ (IKKαβ), p-IKKαβ, p65, p-p65, extracellular signal-regulated kinase (ERK), p-ERK, c-Jun N-terminal kinase (JNK), p-JNK, p38, p-p38, and secondary anti-rabbit antibodies were acquired from Cell Signaling Technology (Beverly, MA, USA). Antibodies against interleukin-1 beta (IL-1β), superoxide dismutase 2 (SOD2), catalase (CAT), β-actin, and secondary anti-mouse antibodies were from Santa Cruz Biotechnology (Santa Cruz, CA, USA). The anti-heme oxygenase-1 (HO-1) antibody was obtained from Abcam (Cambridge, UK). All chemicals used in the experiments were of the highest available grade.

### 2.2. Preparation of Various Extracts from D. polystachya and D. bulbifera

**Figure 1:**
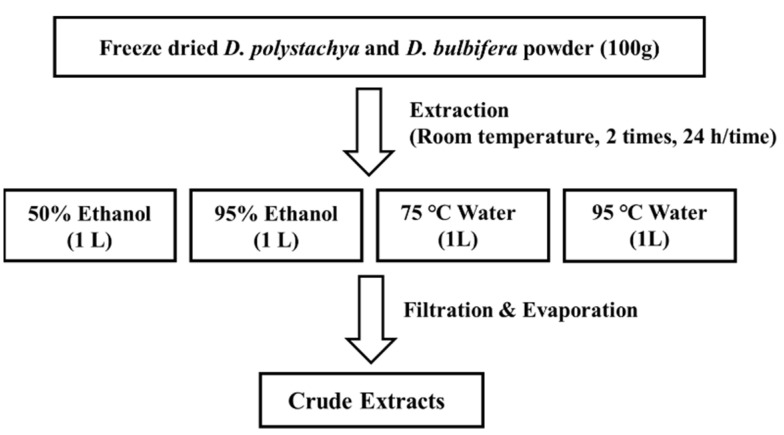
Extraction scheme for preparation of various extracts from *D. polystachya* and *D. bulbifera*.

Yam was cultivated in Andong, Gyeongsangbuk-do. Freeze-dried *D. polystachya* and *D. bulbifera* were soaked in 75 °C, and 95 °C water and in 50%, and 95% EtOH at a ratio of 1:10 (*w*/*v*) for 24 h, then filtered and concentrated to obtain the extract (Figure 1).

### 2.3. Preparation of Yam Mucilage Polysaccharides

**Figure 2:**
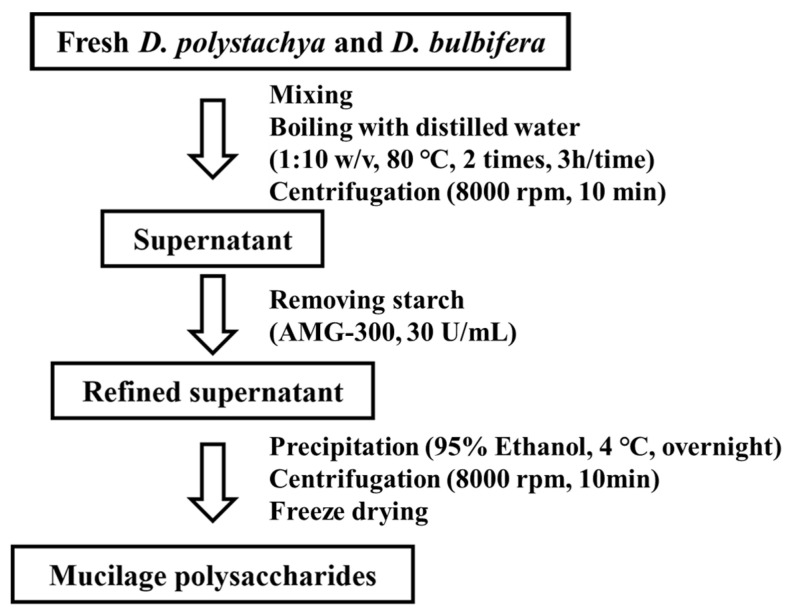
Extraction scheme for preparation of mucilage polysaccharides from *D. polystachya* and *D. bulbifera*.

Yam mucilage polysaccharides were extracted using a modified hot water extraction method [22]. Two kinds of Fresh *D. polystachya* and *D. bulbifera* were blended using a high-speed blender (HC-BL2000 High-speed Blender Axlerim, Happy Call, Gimhae, Republic of Korea) and mixed with distilled water at a ratio of 1:10 (*w*/*v*). The mixture was extracted at 80 °C for 3 h. The extract was centrifuged at 8000 rpm for 10 min to obtain the supernatant. To remove the starch, 30 U/mL of AMG-300 (Novozymes, Bagsværd, Denmark) was added, and the reaction was conducted at 60 °C for 24 h. Following this, 94% ethanol (1:4, *v*/*v*) was added, and the solution was left to precipitate overnight at 4 °C. The precipitate was collected by centrifuging at 8000 rpm for 10 min, and then processed further. It was freeze-dried at −87 °C for 3 days to obtain a yam mucilage polysaccharide extract (Figure 2). The mucilage polysaccharides from the short yam (*D. polystachya*) are abbreviated as DpMP, and those from the round yam (*D. bulbifera*) are abbreviated as DbMP.

### 2.4. Cell Cultures

Murine RAW 264.7 macrophage cells, obtained from the American Type Culture Collection (ATCC, Manassas, VA, USA), were cultured in Dulbecco’s modified Eagle medium (DMEM), supplemented with 100 U/mL of penicillin, 10% fetal bovine serum, and 100 µg/mL of streptomycin, and maintained at 37 °C in a 5% CO_2_ incubator.

The Caco-2 cell line, also acquired from ATCC, was cultivated in a minimum essential medium (MEM), enriched with 100 U/mL of penicillin, 10% fetal bovine serum, and 100 µg/mL of streptomycin, under similar conditions at 37 °C in a 5% CO_2_ environment.

### 2.5. Cell Viability

To evaluate the cell viability of the ethanol extract, water extract, and polysaccharides on the RAW 264.7 cells and Caco-2 cells, the MTT assay was performed. Cells were plated in 96-well plates and treated with samples at different concentrations for 24 h. After treatment, the cells were incubated with an MTT reagent (5 mg/mL) for 3 h at 37 °C in a 5% CO_2_ atmosphere. The resulting formazan crystals were dissolved in dimethyl sulfoxide (DMSO), and absorbance was measured at 570 nm using a microplate reader (BioTek Instruments, Inc., Winooski, VT, USA).

### 2.6. NO Production

Cells were seeded into 96-well plates and treated with ethanol extract, water extract, polysaccharides, or LPS for 12 h. NO concentrations in the culture supernatants were determined using the Griess reaction. Specifically, an equal volume of Griess reagent (composed of 1% sulfanilamide in 5% phosphoric acid and 0.1% naphthyl ethylenediamine hydrochloride) was added to the cell culture medium and incubated at room temperature for 10 min. Absorbance was then measured at 546 nm using a microplate reader (BioTek), and NO levels were calculated based on a standard curve generated from sodium nitrite (NaNO_2_).

### 2.7. Interacellular ROS Formation

RAW 264.7 cells were exposed to the samples for 1 h, followed by treatment with 1 μg/mL of LPS for an additional 6 h. Afterward, the cells were incubated with 20 μM DCFH-DA at 37 °C for 1 h. Following the removal of the probe through washing, the intracellular ROS levels were assessed using a microplate reader (BioTek) at an excitation wavelength of 482/20 nm and an emission wavelength of 528/20 nm.

### 2.8. Phagocytosis Activity

The phagocytic activity of the macrophages was assessed using the neutral red uptake method. After a 12 h incubation period with DpMP, DbMP, or LPS (1 μg/mL) (Sigma), 100 μL of a neutral red solution (0.075% in 10 mM PBS) was added and incubated for 1 h. The supernatant was discarded, and the cells in the 96-well plates were washed three times with PBS to remove any unphagocytized neutral red. A lysis solution (50% ethanol and 1% acetic acid, mixed 1:1, with 200 μL per well) was then added to lyse the cells. After overnight incubation at room temperature, the optical density (OD) was measured at 540 nm using a microplate reader (Biotek).

### 2.9. Western Blot Analysis

The cells were seeded in 6-well plates and treated with the samples for 24 h. After treatment, the cells were washed twice with ice-cold phosphate-buffered saline and lysed using a RIPA buffer (Cell Signaling Technology, Danvers, MA, USA). Protein concentration was determined using the Pierce™ BCA Protein Assay Kit (Thermo Fisher Scientific, Waltham, MA, USA). Equal amounts of protein were separated by sodium dodecyl-sulfate polyacrylamide gel electrophoresis (SDS-PAGE) and transferred to a polyvinylidene fluoride (PVDF) membrane using a semi-dry transfer system (Bio-Rad, Hercules, CA, USA). The membrane was blocked with 5% skim milk at room temperature for 2 h, followed by incubation overnight at 4 °C with primary antibodies. Afterward, secondary antibodies were applied for 3 h at 4 °C, and protein bands were visualized using a luminol-based reagent for Western blotting (Santa Cruz Biotechnology, Dallas, TX, USA).

### 2.10. Physicochemical Properties of DpMP and DbMP

#### 2.10.1. Total Glucose, Protein, and Uronic Acid Contents

The carbohydrate content was assessed using the anthrone–sulfuric acid method. In summary, 20 μL of DpMP and DbMP was mixed with 180 μL of sulfuric acid that had 0.1% anthrone. This mixture was chilled at 4 °C for 10 min and then heated to 100 °C for 20 min. After cooling, the absorbance was recorded at 620 nm, and the carbohydrate content was calculated using glucose standard concentrations.

For the protein content analysis, a BCA protein assay kit (Thermo Scientific, Rockford, IL, USA) was utilized. The absorbance was measured at 562 nm, and the protein content was determined using bovine serum albumin as the reference standard.

The uronic acid content was evaluated using the carbazole assay. In brief, 50 μL of DpMP and DbMP was combined with 200 μL of 80% sulfuric acid containing 25 mM of borax (sodium tetraborate). This mixture was heated at 100 °C for 10 min and then allowed to cool to room temperature for 15 min. Following this, 50 μL of a 0.125% carbazole solution in anhydrous ethanol was added. The mixture was heated again at 100 °C for another 10 min and then cooled to room temperature for 15 min. Absorbance was measured at 550 nm, and the uronic acid content was determined using glucuronic acid as the standard.

#### 2.10.2. Monosaccharide Analysis

The monosaccharide compositions of DpMP and DbMP were analyzed using HPLC. Specifically, 5 mg of DpMP and DbMP were mixed with 3 mL of 2 M Trifluoroacetic acid (TFA), and then an HPLC analysis was performed. The monosaccharide compositions of DpMP and DbMP were confirmed using High-Performance Liquid Chromatography (HPLC) (Ⅱ) (Shimadzu, Republic of Korea) equipped with a Sugar-Pak I column (Ø 6.5 × 300 mm, Waters Co., Milford, MA, USA). A total of 0.01 M of calcium disodium ethylenediaminetetraacetic acid (Ca-EDTA) was used for the mobile phase, and the sample injection volume was set to 20 μL and the flow rate was set to 0.5 mL/min. The column temperature was maintained at 90 °C.

#### 2.10.3. Fourier Transform Infrared Spectroscopy (FT-IR) Analysis

The structures of DpMP and DbMP were analyzed using FT-IR (Nicolet iS5, Thermo Scientific, Waltham, MA, USA) with a scan speed of 0.2 cm/s, a resolution of 4 cm^−1^, and a wavelength range of 4000–400 cm^−1^.

### 2.11. Preparation of Probiotic Strains and Cultures

*Lactobacillus acidophilus* KCTC 3164, *Lactococcus lactis* KCTC 3789, *Lactiplantibacillus plantarum* KCTC 3108, and *Limosilactobacillus reuteri* KCTC 3594 were obtained from the Korean Collection for Type Cultures (KCTC, Jeongeup, Republic of Korea). *Bifidobacterium bifidum* IDCC 4201, *Lactobacillus rhamnosus* IDCC 3201, and *Lactiplantibacillus plantarum* IDCC 3501 were obtained from Ildong Bioscience (Pyeongtaek-si, Korea). The probiotic strains were anaerobically cultured in an MRS broth (BD Difco, Franklin Lakes, NJ, USA) at 37 °C in an incubator (JSSI-100C, JS Research, Gongju-City, Republic of Korea) overnight.

### 2.12. Determining the Effect of DpMP and DbMP on the Growth of Probiotics

To evaluate the optimum DpMP and DbMP levels, probiotics were cultivated by adding 0, 0.5, 1, and 2% *w*/*v* of DpMP to 10 mL of MRS broth, which was then sterilized for 15 min at 121 °C. A total of 7 types of probiotics (1 × 10^8^ CFU/mL) were inoculated at 1% (*v*/*v*). The prepared MRS broth mediums were then inoculated with probiotic bacteria (1% *v*/*v*) and incubated for 0, 1, 3, 6, 12, 18, 24, 36, and 48 h at 37 °C. Then, the growth changes of the inoculated probiotic bacteria were evaluated by measuring the OD value at 600 nm using the microplate reader (BioTek) of the probiotic bacteria inoculation culture. The three replications were performed for measurements.

### 2.13. Analysis of Probiotics Attachment to Intestinal Epithelial Cells

Probiotic bacteria pretreated with DpMP and DbMP were collected by centrifugation at 5000 rpm for 10 min and washed twice with sterile PBS. The bacterial pellet was resuspended in MEM without serum or antibiotics. Approximately 1 × 10^8^ CFU/mL of probiotic bacteria were inoculated onto a monolayer of Caco-2 cells and incubated for 2 h in a 5% CO_2_ incubator. After incubation, the Caco-2 monolayer was rinsed three times with sterile PBS to eliminate non-adherent bacteria. The Caco-2 cells, along with the attached bacteria, were detached using Trypsin-EDTA. The number of attached bacteria was quantified through a colony counting assay on MRS agar plates, with the results expressed as a percentage of the adherent bacteria relative to the initial amount added.

### 2.14. Analysis of Probiotics Inhibition of Attachment to Intestinal Epithelial Cells

An adhesion inhibition assay of harmful bacteria to intestinal epithelial cells, *Escherichia coli* (ATCC #43894), obtained from ATCC, was cultured overnight on LB agar plates. Both the probiotic bacteria and *E. coli* pretreated with DpMP and DbMP were harvested by centrifugation at 5000 rpm for 10 min and washed twice with sterile PBS. The bacterial pellet was resuspended in MEM without serum or antibiotics. Probiotic bacteria and *E. coli*, each at a concentration of 1 × 10^8^ CFU/mL, were co-cultured with a Caco-2 cell monolayer for one hour in a 5% CO_2_ incubator. After incubation, the non-adherent bacteria were removed by washing the cells three times with sterile PBS. The Caco-2 cells, along with the adhered bacteria, were harvested using Trypsin-EDTA. The viable *E. coli* cells attached to the Caco-2 monolayer were quantified via a colony counting assay on LB agar plates. The inhibition rate of *E. coli* was determined by calculating the percentage decrease in *E. coli* adhesion in the presence of each probiotic strain, compared to adhesion without probiotics.

### 2.15. Statistical Analysis

The results were presented as the mean ± the standard deviation of three independent experiments. A statistical analysis was performed using the Prism GraphPad version 8.0 software (San Diego, CA, USA). ANOVA and Tukey’s *t*-test were applied for analysis of variance. * *p* < 0.05, ** *p* < 0.01, and *** *p* < 0.001 values indicate significant differences.

## 3. Results and Discussion

### 3.1. Extraction Yields of D. polystachya and D. bulbifera Using a Different Solvent

The extraction yields for *D. polystachya* and *D. bulbifera* were determined by freeze-drying the extracts obtained through various extraction methods, followed by calculating the dry weight and expressing it as a percentage of the solid content in the powdered samples. For *D. polystachya*, the extraction yields for the 50% ethanol, 95% ethanol, 75 °C water, and 95 °C water extractions were 10.6%, 2.1%, 6.93%, and 6.2%, respectively. In comparison, *D. bulbifera* yielded 8.3%, 1.3%, 4.4%, and 3% under the same extraction conditions. This suggests that the extraction yields for *D. polystachya* and *D. bulbifera* varied depending on the solvent and extraction method, with *D. polystachya* consistently showing higher yields across all the tested conditions (Table 1). Similar studies on other *Dioscorea* species provide context. For example, the aqueous extracts of *D. batatas* prepared by boiling water yielded 11.4% [23], while the maceration of the bulbils of *D. bulbifera* in distilled water resulted in a yield of 3.12% [12]. Additionally, the percentage yield from the aqueous and ethanol extracts for the wild variety of *D. bulbifera* were reported as 14.2% and 7.1%, respectively, while those from the edible variety were 13.8% and 6.4%, respectively [24]. A 96% ethanol extract of *D. alata* yielded 3.32% of the initial substance [25]. Compared to these studies, the results of this research indicate that both *D. polystachya* and *D. bulbifera* exhibit extraction yields that align with or slightly differ from the ranges reported for other *Dioscorea* species, depending on the solvent and extraction method used. The consistently higher yields of *D. polystachya* across all conditions suggest that it may contain a greater abundance of soluble bioactive compounds, while the relatively lower yields of *D. bulbifera* highlight the potential differences in its chemical composition. These findings emphasize the distinct chemical profiles of the two species and their respective potential for applications requiring efficient extraction methods.

### 3.2. Effects of D. polystachya and D. bulbifera on LPS-Stimulated Inflammation in RAW 264.7 Cells

#### 3.2.1. Cell Viability of the *D. polystachya* and *D. bulbifera* Extracts in RAW 264.7 Cells

**Figure 3:**
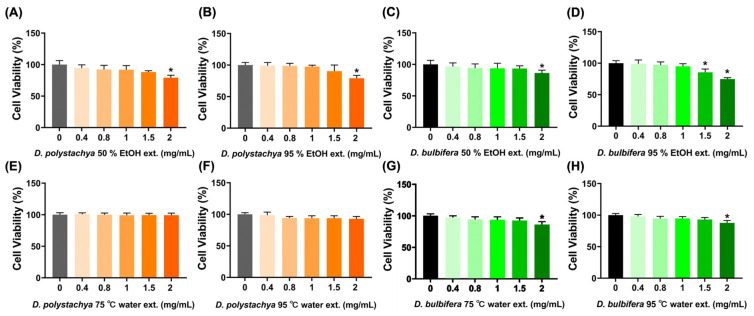
Effects of *D. polystachya* and *D. bulbifera* on cell viability in RAW 264.7 cells. The cells were treated with 0.4, 0.8, 1, 1.5, and 2 mg/mL of the sample for 24 h. Cell viability was determined by MTT assay. (**A**) *D. polystchya* 50% EtOH ext. (**B**) *D. polystachya* 95% EtOH ext. (**C**) *D. bulbifera* 50% EtOH ext. (**D**) *D. bulbifera* 95% EtOH ext. (**E**) *D. polystachya* 75 °C water ext. (**F**) *D. polystachya* 95 °C water ext. (**G**) *D. bulbifera* 75 °C water ext. (**H**) *D. bulbifera* 95 °C water ext. * (*p* ≤ 0.05) were considered significant differences by ANOVA and Tukey’s *t*-test.

The cytotoxicity of the *D. polystachya* and *D. bulbifera* extracts in the RAW 264.7 cells was evaluated using the MTT assay at concentrations of 0.4, 0.8, 1, 1.5, and 2 mg/mL. For *D. polystachya*, the 50% and 95% ethanol extracts showed no significant cytotoxic effects at concentrations below 2 mg/mL, but a significant reduction in cell viability was observed at 2 mg/mL, indicating cytotoxicity (Figure 3A,B). In contrast, the 75 °C and 95 °C water extracts of *D. polystachya* exhibited no cytotoxic effects across all the tested concentrations, maintaining cell viability comparable to the control (Figure 3E,F). For *D. bulbifera*, the 50% ethanol extract demonstrated significant cytotoxicity at 2 mg/mL (Figure 3C), while the 95% ethanol extract showed cytotoxic effects at both 1.5 and 2 mg/mL (Figure 3D), indicating a stronger cytotoxicity compared to the 50% ethanol extract. The 75 °C and 95 °C water extracts of *D. bulbifera* displayed no significant cytotoxicity at concentrations below 2 mg/mL, but a reduction in cell viability was observed at 2 mg/mL (Figure 3G,H). Overall, both the *D. polystachya* and *D. bulbifera* extracts were non-cytotoxic at concentrations below 2 mg/mL for the water extracts and below 1.5 mg/mL for the ethanol extracts. Significant cytotoxic effects were primarily observed at the highest concentration of 2 mg/mL, particularly in the ethanol extracts. Based on these findings, concentrations below 1 mg/mL were selected for subsequent experiments to ensure non-cytotoxic conditions.

#### 3.2.2. Inhibition of ROS Suppression in LPS-Induced RAW 264.7 Cells

**Figure 4:**
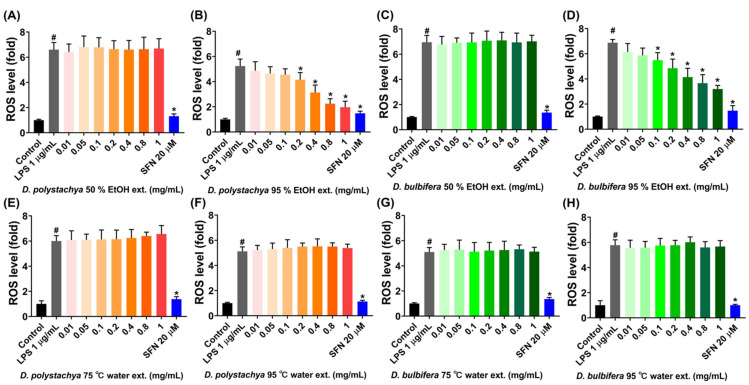
Effects of *D. polystachya* and *D. bulbifera* on ROS formation in LPS-stimulated RAW 264.7 cells. The cells were treated with 0.01, 0.05, 0.1, 0.2, 0.4, 0.8, and 1 mg/mL of the samples for 1 h prior to LPS (1 μg/mL) incubation for an additional 6 h. SFN (20 μM) was used as a positive control. ROS level was measured using the DCFH-DA assay. (**A**) *D. polystchya* 50% EtOH ext. (**B**) *D. polystachya* 95% EtOH ext. (**C**) *D. bulbifera* 50% EtOH ext. (**D**) *D. bulbifera* 95% EtOH ext. (**E**) *D. polystachya* 75 °C water ext. (**F**) *D. polystachya* 95 °C water ext. (**G**) *D. bulbifera* 75 °C water ext. (**H**) *D. bulbifera* 95 °C water ext. # *p* < 0.05 vs. control and * *p* < 0.05 vs. LPS 1 μg/mL were considered significant differences by ANOVA and Tukey’s *t*-test.

The abilities of *D. polystachya* and *D. bulbifera* extracts to inhibit LPS-induced ROS production in RAW 264.7 macrophage cells were assessed using DCFH-DA assays. ROS generation is a hallmark of oxidative stress, which can lead to cellular damage and exacerbate inflammation by affecting macromolecules like proteins, lipids, and DNA. Elevated ROS levels are commonly observed during acute and chronic inflammation due to the activation of immune and inflammatory cells [26]. In this study, the RAW 264.7 cells were treated with extract concentrations of 0.01, 0.05, 0.1, 0.2, 0.4, 0.8, and 1 mg/mL, with sulforaphane (20 μM) used as a positive control. The LPS-treated group exhibited a significant increase in ROS levels compared to the untreated control, confirming an inflammatory response. A treatment with the 95% ethanol extract of *D. polystachya* significantly reduced ROS production in a dose-dependent manner, with notable inhibitory effects observed at concentrations of 0.2 mg/mL and higher (Figure 4B). Similarly, the 95% ethanol extract of *D. bulbifera* demonstrated significant ROS suppression starting at 0.1 mg/mL, with further reductions at higher concentrations (Figure 4D). Conversely, the 50% ethanol extracts from both yam species, as well as their water extracts prepared at 75 °C and 95 °C, did not show significant inhibitory effects on ROS production at any tested concentration (Figure 4A,C,E–H). These findings suggest that the 95% ethanol extracts are particularly effective in mitigating oxidative stress, likely due to the enhanced extraction and concentration of the specific bioactive compounds with antioxidant properties. These results underscore the potential of 95% ethanol extracts of *D. polystachya* and *D. bulbifera* as potent inhibitors for suppressing LPS-induced ROS generation, highlighting their applicability for managing oxidative stress and inflammation.

#### 3.2.3. Inhibition of NO Production in LPS-Induced RAW 264.7 Cells

**Figure 5:**
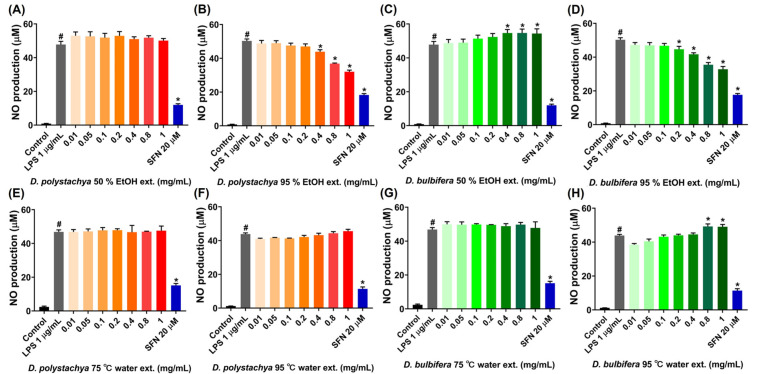
Effects of *D. polystachya* and *D. bulbifera* on NO production in LPS-stimulated RAW 264.7 cells. The cells were treated with 0.01, 0.05, 0.1, 0.2, 0.4, 0.8, and 1 mg/mL of samples for 1 h prior to LPS (1 μg/mL) incubation for an additional 24 h. SFN (20 μM) was used as a positive control. NO production was determined by Griess assay. (**A**) *D. polystachya* 50% EtOH ext. (**B**) *D. polystachya* 95% EtOH ext. (**C**) *D. bulbifera* 50% EtOH ext. (**D**) *D. bulbifera* 95% EtOH ext. (**E**) *D. polystachya* 75 °C water ext. (**F**) *D. polystachya* 95 °C water ext. (**G**) *D. bulbifera* 75 °C water ext. (**H**) *D. bulbifera* 95 °C water ext. # *p* < 0.05 vs. Control and * *p* < 0.05 vs. LPS 1 μg/mL was considered as significant differences by ANOVA and Tukey’s *t*-test.

The inhibitory effects of the *D. polystachya* and *D. bulbifera* extracts on LPS-induced NO production in the RAW 264.7 macrophage cells were assessed. NO is a critical inflammatory mediator, and excessive production during inflammatory responses can lead to cellular and tissue damage or induce DNA mutations [27]. The RAW 264.7 cells were treated with various concentrations (0.01, 0.05, 0.1, 0.2, 0.4, 0.8, and 1 mg/mL) of the extracts, with sulforaphane (20 μM) serving as the positive control. LPS stimulation significantly increased the NO levels compared to the untreated control, demonstrating the induction of an inflammatory response. The 95% ethanol extract of *D. polystachya* showed a dose-dependent reduction in NO production, with significant inhibitory effects observed at concentrations of 0.4 mg/mL and higher (Figure 5B). Similarly, the 95% ethanol extract of *D. bulbifera* effectively inhibited NO production, with significant reductions observed starting at 0.2 mg/mL and increasing at higher concentrations (Figure 5D). In contrast, the 50% ethanol extracts of both species, as well as their water extracts prepared at 75 °C and 95 °C, did not exhibit significant inhibitory effects on NO production at any tested concentration (Figure 5A,C,E–H). These results indicate that the 95% ethanol extracts of *D. polystachya* and *D. bulbifera* possess potent anti-inflammatory activity by reducing NO production in a dose-dependent manner. This difference in extract activity may be linked to the solubility of specific bioactive phytochemicals, such as flavonoids and saponins, which are known to have anti-inflammatory properties and are better extracted at higher ethanol concentrations [28]. Additionally, the dose-dependent inhibition observed in the 95% ethanol extracts further supports their therapeutic potential for regulating inflammation through NO production.

#### 3.2.4. Anti-Inflammatory Effects of *D. polystachya* and *D. bulbifera* in LPS-Stimulated RAW 264.7 Cells

**Figure 6:**
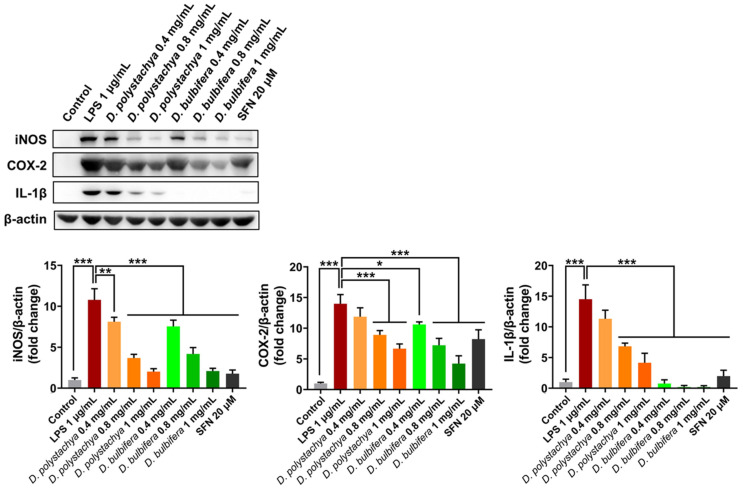
Effects of *D. polystachya* and *D. bulbifera* on the protein expression of pro-inflammatory mediators in LPS-stimulated RAW 264.7 cells. The cells were pretreated with 0.4, 0.8, and 1 mg/mL of *D. polystachya* and *D. bulbifera* for 1 h prior to LPS (1 μg/mL) incubation for an additional 24 h. Expression of iNOS, COX-2, and IL-1β was determined by Western blot. SFN (20 μM) was used as a positive control. * (*p* ≤ 0.05), ** (*p* ≤ 0.01), and *** (*p* ≤ 0.001) were considered significant differences by ANOVA and Tukey’s *t*-test.

The anti-inflammatory effects of the 95% ethanol extracts from *D. polystachya* and *D. bulbifera* were evaluated by analyzing the expression of pro-inflammatory mediator proteins, including iNOS, COX-2, and IL-1β, in LPS-stimulated RAW 264.7 macrophage cells. An excessive production of these mediators is a hallmark of chronic inflammation and contributes to the development of various inflammatory diseases. iNOS is a key enzyme in an inflammatory response, synthesizing NO from arginine, while COX-2 catalyzes the production of PGE2 (prostaglandin E2), a potent inflammatory cytokine. IL-1β, a critical mediator of chronic inflammation, is also implicated in inflammation-related tumorigenesis [29]. The inhibitory effects of the extracts on nitric oxide and reactive oxygen species were initially confirmed, followed by an assessment of their impact on inflammatory mediator protein expression through a Western blot analysis. The results demonstrated a significant, dose-dependent suppression of iNOS, COX-2, and IL-1β protein expression by the 95% ethanol extracts of both *D. polystachya* and *D. bulbifera* (Figure 6). Notably, the 95% ethanol extract of *D. bulbifera* showed greater efficacy in inhibiting the expression of these inflammatory proteins compared to the extract from *D. polystachya*. At a concentration of 1 mg/mL, the *D. bulbifera* extract exhibited superior inhibitory effects, indicating its stronger anti-inflammatory potential. The observed suppression of inflammatory protein expression suggests that the 95% ethanol extracts from both yam species possess significant anti-inflammatory properties. The dose-dependent inhibition of inflammatory proteins further underscores the therapeutic potential of these extracts. These findings highlight the significant anti-inflammatory potential of the 95% ethanol extracts of *D. polystachya* and *D. bulbifera*, particularly the superior activity of *D. bulbifera*.

### 3.3. Inhibition of the MAPK and NF-κB Signaling Pathways by D. polystachya and D. bulbifera Extracts in RAW 264.7 Cells

#### 3.3.1. Effects on the MAPK Signaling Pathways in RAW 264.7 Cells

**Figure 7:**
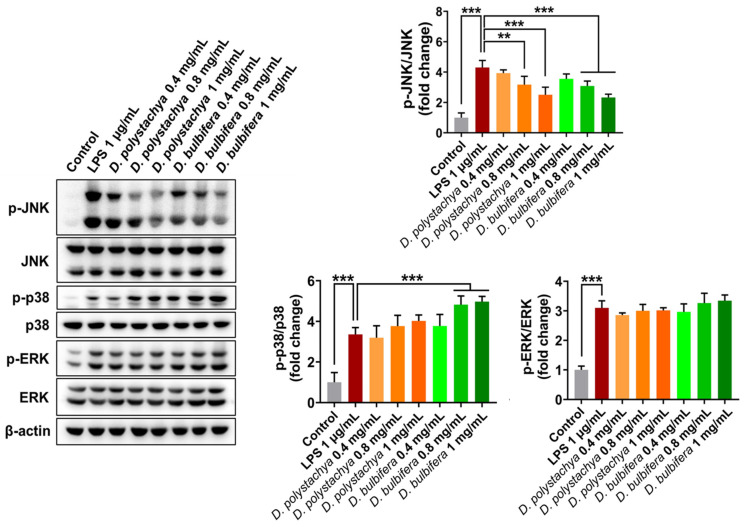
Effects of *D. polystachya* and *D. bulbifera* on the protein expression of MAPK pathway in LPS-stimulated RAW 264.7 cells. The cells were pretreated with 0.4, 0.8, and 1 mg/mL of *D. polystachya* and *D. bulbifera* for 1 h prior to LPS (1 μg/mL) incubation for an additional 1 h. Expressions of JNK, p38, and ERK were determined by Western blot. ** (*p* ≤ 0.01), and *** (*p* ≤ 0.001) were considered significant differences by ANOVA and Tukey’s *t*-test.

The anti-inflammatory effects of the 95% ethanol extracts from *D. polystachya* and *D. bulbifera* were further evaluated through their impact on the activation of the MAPK signaling pathway, which is a major regulator of inflammatory mediators. MAPKs, a group of serine/threonine protein kinases, play a crucial role in cellular responses to external stress and various biological functions. Specifically, the JNK and p38 pathways are key regulators in the production of inflammatory mediators [30]. The stimulation of RAW 264.7 macrophage cells with LPS resulted in significantly increased phosphorylation levels of MAPK components, including ERK, JNK, and p38. Treatment with the 95% ethanol extracts of *D. polystachya* and *D. bulbifera* at concentrations of 0.4, 0.8, and 1 mg/mL dose-dependently suppressed the phosphorylation of JNK (p-JNK), indicating effective inhibition of this signaling component (Figure 7). This suggests the strong inhibitory effect of the extracts on JNK phosphorylation. In contrast, the extracts did not show inhibitory effects on the LPS-induced phosphorylation of p38 or ERK, suggesting that their anti-inflammatory activity is specific to the inhibition of JNK activation within the MAPK pathway. These findings demonstrate that the anti-inflammatory properties of the 95% ethanol extracts from both yam species are mediated through the suppression of JNK phosphorylation. The selective targeting of JNK in the MAPK pathway highlights the potential therapeutic relevance of these extracts in managing the inflammatory conditions driven by JNK activation.

#### 3.3.2. Effects on NF-κB Signaling Pathways in RAW 264.7 Cells

**Figure 8:**
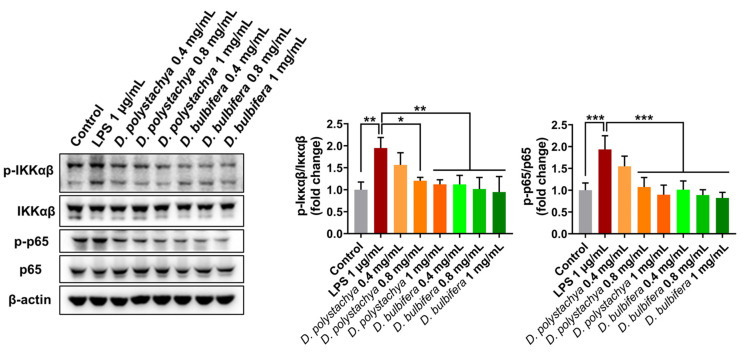
Effects of *D. polystachya* and *D. bulbifera* on the protein expression of NF-κB pathway in LPS-stimulated RAW 264.7 cells. The cells were pretreated with 0.4, 0.8, and 1 mg/mL of *D. polystachya* and *D. bulbifera* for 1 h prior to LPS (1 μg/mL) incubation for an additional 1 h. Expression of IKKαβ, p65 was determined by Western blot. * (*p* ≤ 0.05), ** (*p* ≤ 0.01), and *** (*p* ≤ 0.001) were considered significant differences by ANOVA and Tukey’s *t*-test.

The effects of the 95% ethanol extracts from *D. polystachya* and *D. bulbifera* on the NF-κB signaling pathway, a key regulator of inflammatory mediator expression, were evaluated. In the RAW 264.7 macrophage cells stimulated with LPS, the activation of IKK leads to its phosphorylation and the subsequent nuclear translocation of NF-κB (p65), which induces the expression of inflammation-related genes. LPS treatment significantly increased the phosphorylation levels of IKKαβ and p65 in the RAW 264.7 cells, indicating a robust activation of the NF-κB signaling pathway. Treatment with the 95% ethanol extracts of *D. polystachya* and *D. bulbifera* at concentrations of 0.4, 0.8, and 1 mg/mL significantly suppressed the phosphorylation of both IKKαβ and p65 in a dose-dependent manner (Figure 8). These findings suggest that both extracts effectively inhibit the LPS-induced activation of the NF-κB signaling pathway. The suppression of NF-κB activation highlights the anti-inflammatory potential of these extracts, as NF-κB is a critical transcription factor involved in the regulation of pro-inflammatory cytokines, chemokines, and enzymes. The observed inhibition of p-IKKαβ and p-p65 suggests that the 95% ethanol extracts of *D. polystachya* and *D. bulbifera* exert their anti-inflammatory effects by targeting the NF-κB signaling pathway [31]. This supports the therapeutic potential of these extracts in managing inflammation-associated conditions through the modulation of key signaling pathways.

#### 3.3.3. Role of the MAPK/JNK Signaling Pathway in LPS-Induced RAW 264.7 Cells

**Figure 9:**
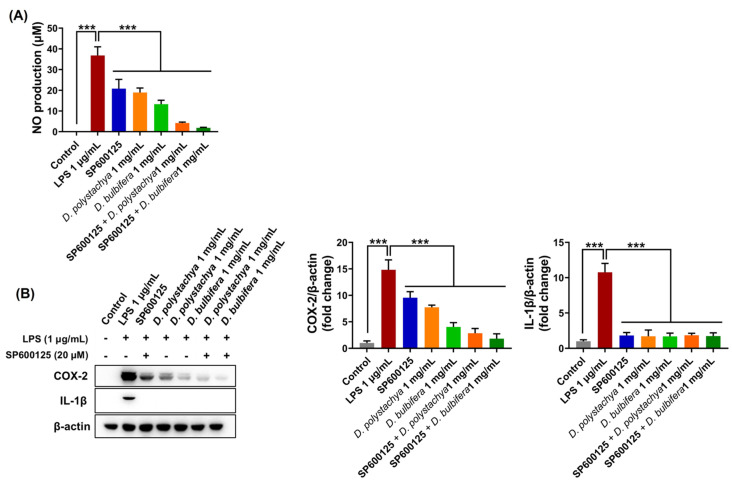
Effects of *D. polystachya* and *D. bulbifera* on NO production (**A**) and protein expression of pro-inflammatory cytokines (**B**) through JNK MAPK signaling pathway in LPS-stimulated RAW 264.7 cells. The cells were pretreated with 1 mg/mL of *D. polystachya* and *D. bulbifera* or JNK inhibitor (SP600125) for 1 h prior to LPS (1 μg/mL) incubation for an additional 12 h. NO production was determined by Griess assay. Expressions of COX-2 and IL-1β were determined by Western blot. *** (*p* ≤ 0.001) was considered significant differences by ANOVA and Tukey’s *t*-test.

To further investigate the role of the JNK MAPK signaling pathway in the anti-inflammatory effects of the 95% ethanol extracts from *D. polystachya* and *D. bulbifera*, the RAW 264.7 macrophage cells were pretreated with the extracts (1 mg/mL) or SP600125, a specific JNK inhibitor (20 μM), for 1 h prior to LPS stimulation. SP600125 significantly inhibited LPS-induced NO production, demonstrating comparable inhibitory effects to those observed with the 95% ethanol extracts of both yam species (Figure 9A). Additionally, treatment with SP600125 resulted in a marked reduction in the expression levels of COX-2 and IL-1β, key inflammatory mediators, similar to the effects of the yam extracts (Figure 9B). These findings confirmed that both cytokines are regulated by the JNK signaling pathway. The results indicate that the inhibition of the JNK signaling pathway effectively prevents LPS-induced inflammatory responses. The similarity between the effects of the JNK inhibitor and the 95% ethanol extracts of *D. polystachya* and *D. bulbifera* suggests that the anti-inflammatory activity of the extracts is mediated, at least in part, by targeting the JNK MAPK pathway. This highlights the critical role of JNK inhibition in mitigating the expression of pro-inflammatory mediators and underscores the therapeutic potential of these extracts in managing inflammation-driven conditions.

### 3.4. Physicochemical Characterization of Yam Mucilage Polysaccharides

#### 3.4.1. Chemicals Properties of DpMP and DbMP

The chemical properties of the mucilage polysaccharides (DpMP and DbMP) extracted from *D. polystachya* and *D. bulbifera* were analyzed, including their yields and the contents of total carbohydrates, uronic acid, and protein (Table 2). The yield of DpMP was 0.93 ± 0.31%, with the contents of carbohydrates, protein, and uronic acid being 26.44 ± 1.21%, 20.83 ± 1.51%, and 19.18 ± 0.93%, respectively. In contrast, DbMP showed a higher yield of 4.05 ± 0.96%, with the carbohydrate, protein, and uronic acid contents measured at 22.16 ± 1.47%, 23.43 ± 1.50% and 13.48 ± 0.51%, respectively. These results highlight the compositional differences between the two yam-derived polysaccharides, with DbMP exhibiting a notably higher yield compared to DpMP, while DpMP showed slightly higher carbohydrate and uronic acid contents. These variations may reflect differences in the structural and functional properties of the polysaccharides.

#### 3.4.2. Monosaccharide Composition of DpMP and DbMP

**Figure 10:**
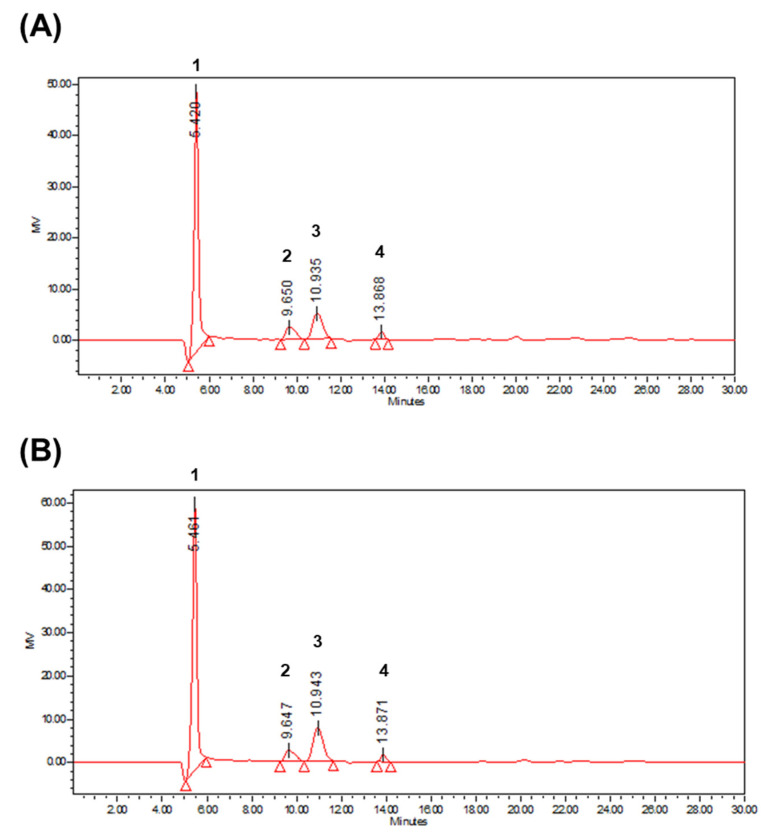
HPLC chromatograms of yam polysaccharides. (**A**) DpMP, and (**B**) DbMP. 1-galacturonic acid, 2-glucose, 3-galactose, 4-mannose.

The investigation of the structure–activity relationships in plant polysaccharides underscores the significance of monosaccharide composition, as it plays a crucial role in determining the biological activity of these polysaccharides [32]. An analysis of the monosaccharide composition of the mucilage polysaccharides (DpMP and DbMP) extracted from *D. polystachya* and *D. bulbifera* using HPLC revealed that both are primarily composed of galacturonic acid, glucose, galactose, and mannose (Figure 10). Notably, galacturonic acid has a high proportion. This high galacturonic acid content suggests that both polysaccharides are rich in uronic acids, which are often associated with biological activities such as immune modulation and antioxidant properties [33,34]. Additionally, the presence of glucose and galactose enhances their structural stability and functional versatility, further supporting their potential bioactivity [35,36]. Specifically, DpMP predominantly consists of galacturonic acid (69.98%), galactose (18.38%), glucose (8.84%), and mannose (2.80%) indicating these monosaccharides as its core structural components. DbMP exhibits a similar profile, highlighting comparable biological and functional properties between the two polysaccharides (Table 3). This compositional resemblance suggests that their biological activities may be interconnected and influenced by their shared monosaccharide structures. These findings underscore the importance of galacturonic acid and the associated monosaccharides in defining the structure and bioactivity of DpMP and DbMP.

#### 3.4.3. FT-IR Spectrum Analysis

**Figure 11:**
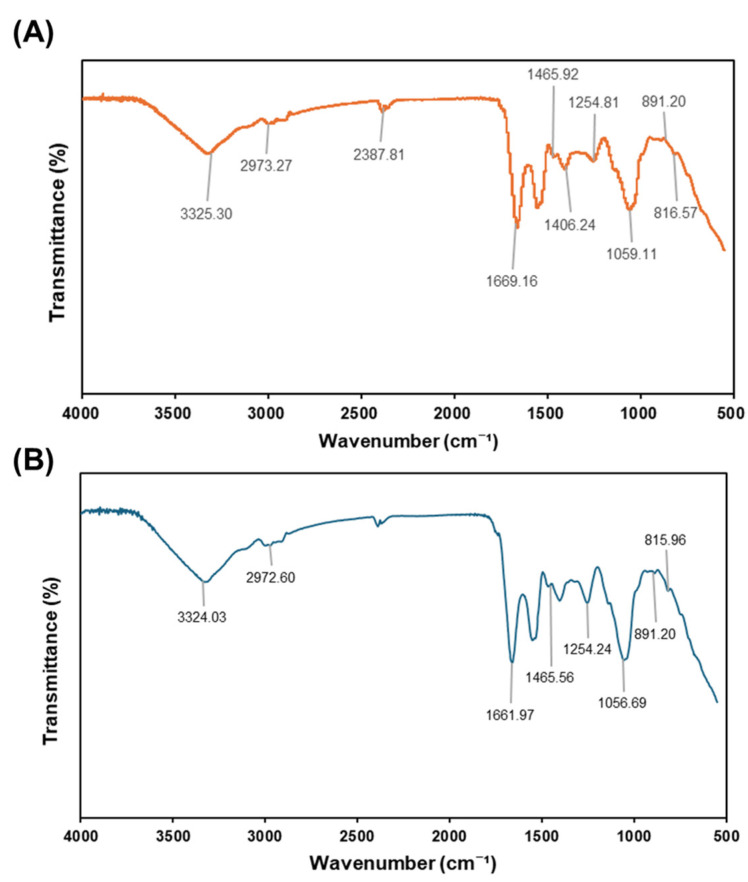
FT-IR spectrum of yam polysaccharides. (**A**) DpMP, and (**B**) DbMP.

The FT-IR spectra of the yam polysaccharides (Figure 11) revealed distinct absorption peaks that correspond to characteristic functional groups of polysaccharides, highlighting their complex chemical structures [37]. A strong and broad absorption band observed around 3325 cm^−1^ (*D. polystachya*) and 3324 cm^−1^ (*D. bulbifera*) indicates the presence of hydroxyl (-OH) groups, which are key structural features contributing to the hydrophilicity of polysaccharides. Peaks at 2973 cm^−1^ (*D. polystachya*) and 2972 cm^−1^ (*D. bulbifera*) represent the asymmetric and symmetric stretching vibrations of aliphatic C-H groups, confirming the carbohydrate nature of the polysaccharides. A sharp peak near 1669–1661 cm^−1^ suggests the stretching of carbonyl (C=O) groups, which are typically associated with uronic acids, a component commonly found in plant polysaccharides. Additional peaks in the range of 1465–1406 cm^−1^, correspond to the bending vibrations of C-H bonds and asymmetric stretching of C-O groups, which are essential to the polysaccharide backbone structure. Furthermore, the peaks observed at 1254 cm^−1^, 1059 cm^−1^, and 891–815 cm^−1^ confirm the presence of glycosidic linkages (C-O-C stretching), specifically β-glycosidic bonds, which are characteristic of polysaccharides. Overall, the FT-IR spectra of yam polysaccharides confirmed the presence of key functional groups, including hydroxyl, carbonyl, and glycosidic linkages, which highlight their structural intricacies and molecular organization. These structural features not only underscore the characteristic architecture of polysaccharides but also provide valuable insights into their chemical composition and potential bioactivity, further supporting their role in various biological applications.

### 3.5. Immuno-Enhancing and Antioxidant Effect of DpMP and DbMP in RAW 264.7 Cells

#### 3.5.1. Cell Viability of DpMP and DbMP in RAW 264.7 Cells

**Figure 12:**
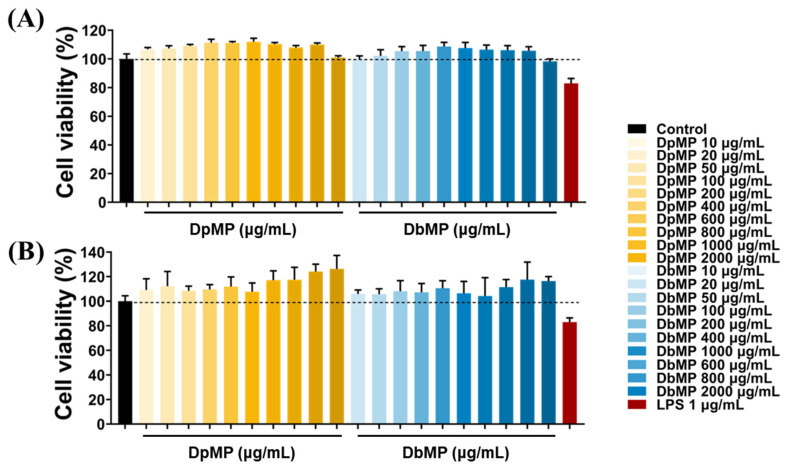
Effects of DpMP and DbMP on cell viability in (**A**) Raw 264.7, and (**B**) Caco-2. The cells were treated with DpMP and DbMP (10–2000 μg/mL), or LPS (1 μg/mL) for 24 h. The cell viability was determined by MTT assay. Results are expressed as mean ± SD.

The cytotoxicity of the mucilage polysaccharides (DpMP and DbMP) from *D. polystachya* and *D. bulbifera* was evaluated in the RAW 264.7 and Caco-2 cells using the MTT assay after 24 h of treatment with varying concentrations (10–2000 μg/mL) (Figure 12). The results indicated no significant reduction in cell viability at any tested concentration compared to the control group, demonstrating the absence of cytotoxic activity for both DpMP and DbMP under these conditions. These findings confirm that DpMP and DbMP are non-toxic to RAW 264.7 macrophage cells and Caco-2 intestinal epithelial cells, even at the highest tested concentration of 2000 μg/mL. This establishes the safety of DpMP and DbMP for further experimental evaluations. The lack of cytotoxic effects highlights the feasibility of employing these concentrations in future studies to investigate their bioactivity and therapeutic applications.

#### 3.5.2. Immuno-Enhancing Effect of DpMP and DbMP in RAW 264.7 Cells

RAW 264.7 macrophage cells are a widely used model for studying innate immunity and immune regulation. These cells play a pivotal role in the body’s defense against infections and inflammation by producing ROS, NO, and cytokines such as TNF-α, IL-6, and IL-1β [38]. These immune mediators coordinate and amplify the immune response. Polysaccharides are known to mediate their immune-regulatory effects by modulating macrophage activity, impacting immune responses through various mechanisms [39]. To investigate the immuno-enhancing effects of DpMP and DbMP in RAW264.7 cells, the cells were treated with concentrations ranging from 10 to 2000 µg/mL for 6 h (for ROS) and from 10 to 400 µg/mL for 12 h (for NO, phagocytosis, and cytokines). LPS at 1 μg/mL was used as a positive control, and the immuno-enhancing effects were evaluated using neutral red assays, a Western blot analysis, and functional measurements of ROS and NO production. ROS plays a critical role in immune regulation by promoting the expression of cytokines and stimulating the secretion of immunomodulators. These molecules are essential for coordinating the body’s defense against pathogens and controlling inflammation, making ROS integral to the immune system’s ability to respond effectively to infections and maintain overall immune balance [40,41]. Phagocytosis is a fundamental mechanism in innate immunity, enabling macrophages to engulf and eliminate pathogens [42]. NO, synthesized from L-arginine by iNOS, serves as a critical effector molecule in immune responses. Primarily produced by activated macrophages, NO plays a pivotal role in the cytotoxic and cytostatic mechanisms of innate immunity, aiding in the inhibition of infectious pathogens. NO also contributes to various physiological functions, such as maintaining vascular homeostasis and mediating neurotransmissions. While excessive NO production can act as a mediator in inflammatory diseases, the NO secreted by activated immune cells under normal conditions functions as a signaling molecule, enhancing resistance to pathogens [43]. The iNOS oxidizes L-arginine to produce L-citrulline and NO, a key signaling molecule in immune responses. Adequate NO secretion by the activated immune cells functions as an immune signaling mediator, stimulating immune cell activation and enhancing immune responses [44]. Additionally, COX-2, which is induced upon stimulation, further amplifies immune competence by facilitating the production of inflammatory mediators. When macrophages are activated, they also secrete cytokines such as IL-1β and TNF-α, which play essential roles in regulating immune and inflammatory responses [45,46].

**Figure 13:**
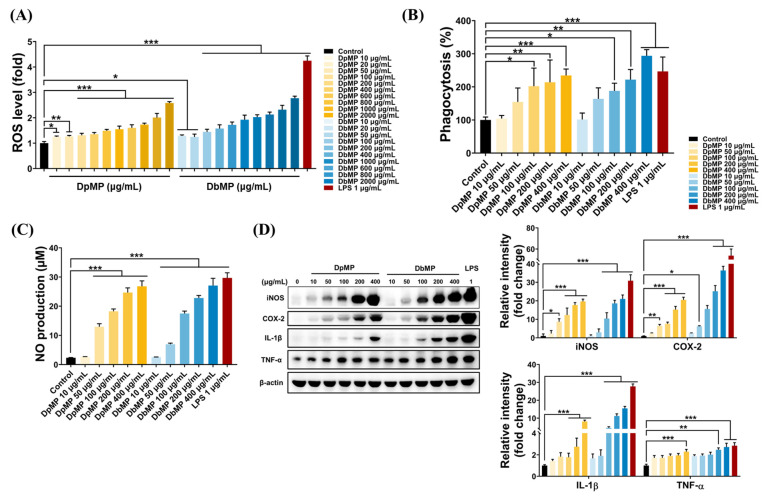
Effects of DpMP and DbMP on immune-enhancing activities in RAW 264.7 cells. RAW 264.7 cells were treated with DpMP and DbMP (10–2000 μg/mL), or LPS (1 μg/mL) for 8 h (ROS) and DpMP and DbMP (10–400 μg/mL) or LPS (1 μg/mL) for 12 h (phagocytosis, NO, cytokines). (**A**) ROS formation. (**B**) Phagocytosis. (**C**) NO production. (**D**) Protein expressions of iNOS, COX-2, IL-1β, and TNF-α. LPS served as a positive control. Results are expressed as mean ± SD. Statistical significance was assessed using one-way or two-way ANOVA, followed by Tukey’s post-hoc test. Significance levels were set at * *p* < 0.05, ** *p* < 0.01, and *** *p* < 0.001.

DpMP and DbMP significantly elevated ROS production in the RAW264.7 cells. The increased ROS levels demonstrate the capacity of these polysaccharides to stimulate macrophage function, thereby reinforcing their potential as immuno-enhancing agents (Figure 13A). The phagocytosis of the neutral red assay demonstrated that both DpMP and DbMP significantly increased the phagocytic activity of the RAW 264.7 cells in a dose-dependent manner (Figure 13B). This result underscores the potential of these polysaccharides to enhance innate immune defense mechanisms. Treatment with DpMP and DbMP significantly increased NO production in the RAW 264.7 cells in a dose-dependent manner, akin to the effect observed with LPS treatment (Figure 13C). This elevation in NO highlights the immuno-enhancing properties of these polysaccharides, emphasizing their role in macrophage activation. The Western blot analysis revealed a dose-dependent increase in the protein expression of these inflammatory mediators following treatment with DpMP and DbMP (Figure 13D). These results indicate that DpMP and DbMP have immuno-enhancing properties through the activation of macrophages.

### 3.6. Antioxidant Activities of DpMP and DbMP in RAW 264.7 Cells

**Figure 14:**
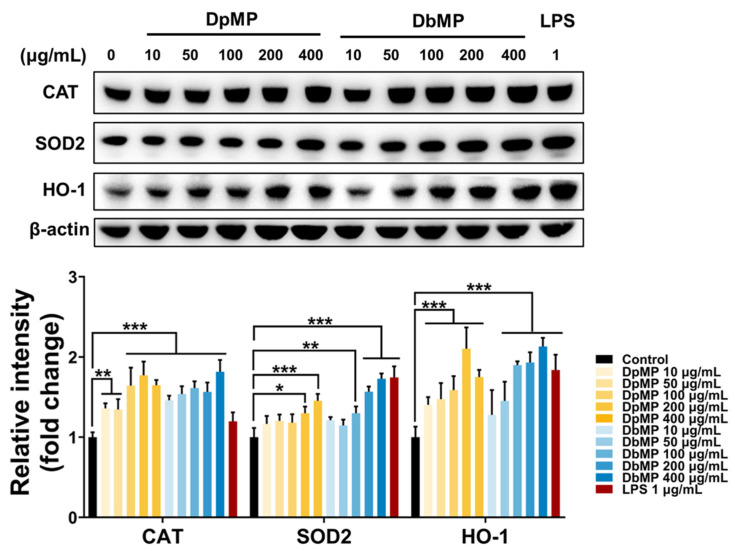
Effects of DpMP and DbMP on antioxidant activities in RAW 264.7 cells. The cells were treated with DpMP and DbMP (10, 50, 100, 200, and 400 μg/mL), or LPS (1 μg/mL) for 12 h. Protein expressions of CAT, SOD2, and HO-1. Results are expressed as mean ± SD. Statistical significance was assessed using one-way or two-way ANOVA, followed by Tukey’s post-hoc test. Significance levels were set at * *p* < 0.05, ** *p* < 0.01, and *** *p* < 0.001.

Cells protect themselves from oxidative stress by eliminating excess oxidants or by enhancing the activity of antioxidant enzymes. These enzymes include CAT and SOD2, which are primary antioxidants that catalyze the decomposition of ROS, and HO-1, a phase II enzyme essential for detoxifying reactive compounds and modulating antioxidant gene expression [47,48]. The effects of DpMP and DbMP on the expression of these antioxidant enzymes in RAW 264.7 macrophage cells were evaluated via Western blot analysis. CAT and SOD2 are essential antioxidant enzymes that alleviate oxidative stress through the catalysis of reactive oxygen species decomposition. In contrast, HO-1 is crucial for regulating antioxidant gene expression and the detoxification of electrophilic compounds [49]. Both DpMP and DbMP significantly enhanced the expression of HO-1 in a dose-dependent manner, indicating an activation of the antioxidant signaling pathways. Similarly, the expression levels of CAT and SOD2 were markedly increased following treatment with DpMP and DbMP, demonstrating their ability to decompose hydrogen peroxide and prevent the formation of free radicals (Figure 14). These findings suggest that DpMP and DbMP effectively enhance the cellular antioxidant defense system by upregulating both primary and phase II antioxidant enzymes. This enhancement reflects their potential in mitigating oxidative stress, which plays a critical role in maintaining cellular homeostasis and serving as a protective response against oxidative damage-induced diseases.

### 3.7. Prebiotic Effects of DpMP and DbMP in Probiotics

#### 3.7.1. Effects on the Growth of Beneficial Probiotics

**Figure 15:**
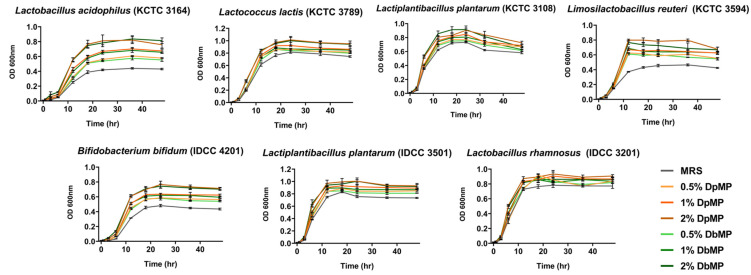
Bacterial growth curves of *Lactobacillus* strains and *Bifidobacterium* strains in MRS broth with 0%, 0.5%, 1%, and 2% of DpMP and DbMP after incubation measured by turbidity (optical density, 600 nm). Tested bacterial strains were *L. acidophilus* (KCTC 3164), *L. lactis* (KCTC 3789), *L. plantarum* (KCTC 3108), *L. reuteri* (KCTC 3594), *B. bifidum* (IDCC 4201), *L. plantarum* (IDCC 3501), and *L. rhamnosus* (IDCC 3201). Data are presented as the mean ± SD.

The growth-promoting effects of DpMP and DbMP on the probiotics were evaluated using seven probiotic strains, including *L. acidophilus* (KCTC 3164), *L. lactis* (KCTC 3789), *L. plantarum* (KCTC 3108), *L. reuteri* (KCTC 3594), *B. bifidum* (IDCC 4201), *L. plantarum* (IDCC 3501), and *L. rhamnosus* (IDCC 3201). The probiotics were cultured in an MRS broth supplemented with 0.5%, 1%, or 2% DpMP or DbMP, and the growth curves were monitored over 48 h. The MRS broth without polysaccharide supplementation was used as the control. Both DpMP and DbMP significantly promoted the growth of all the tested probiotics in a concentration-dependent manner, with the 2% concentration showing the most pronounced effects. The enhanced growth rates were evident as early as 12 h into the incubation period and sustained throughout the 48 h observation period (Figure 15). Specifically, DpMP and DbMP supplementation markedly improved the OD 600 values of the probiotic cultures compared to the control group, highlighting their prebiotic potential. These results demonstrate the ability of DpMP and DbMP to serve as effective prebiotics, supporting the proliferation of beneficial gut microbiota and potentially improving gut health.

#### 3.7.2. Effects on Gut Epithelial Adhesion of Probiotics and Protection Against Enteropathogens

**Figure 16:**
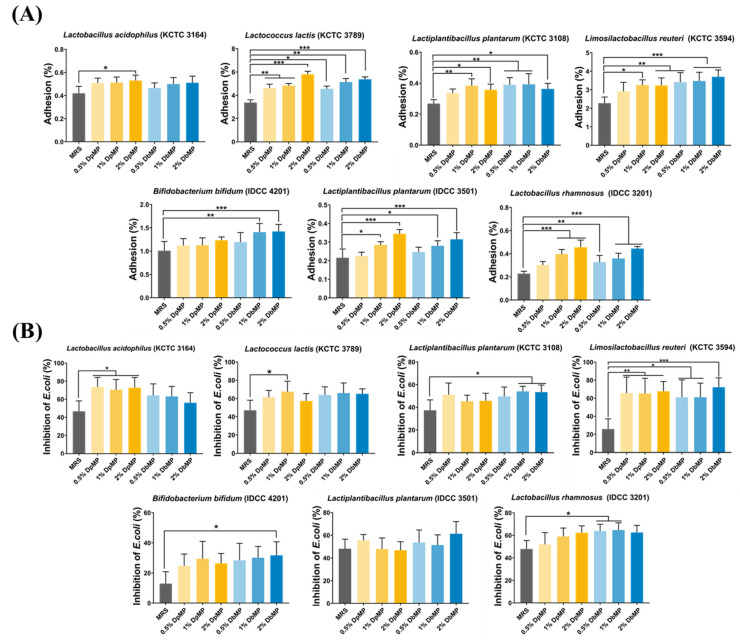
Gut epithelial adhesion (**A**) and inhibition of *E. coli* attachment (**B**) of probiotics. Tested bacterial strains were *L. acidophilus* (KCTC 3164), *L. lactis* (KCTC 3789), *L. plantarum* (KCTC 3108), *L. reuteri* (KCTC 3594), *B. bifidum* (IDCC 4201), *L. plantarum* (IDCC 3501), and *L. rhamnosus* (IDCC 3201). Results are expressed as mean ± SD. Statistical significance was assessed using one-way or two-way ANOVA, followed by Tukey’s post-hoc test. Significance levels were set at * *p* < 0.05, ** *p* < 0.01, and *** *p* < 0.001.

Caco-2 cells, which are gut epithelial cells, exhibit features closely resembling human intestinal cells, including the ability to differentiate, polarize, and form tight junctions [50]. As such, they serve as a widely recognized model for investigating probiotic and pathogen interactions with the intestinal epithelium, particularly adherence mechanisms [51]. Probiotic strains are known to inhibit pathogen attachment by preventing colonization through competitive exclusion. This adhesion inhibition can be attributed to various mechanisms, including antimicrobial compound production by lactic acid bacteria (LAB), competition for receptors and substrates on eukaryotic cells, the modulation of the intestinal mucosal barrier and immune system, and co-aggregation [52]. Polysaccharides play a pivotal role in maintaining intestinal health by promoting probiotic growth and inhibiting the adhesion and proliferation of pathogenic microbes [53]. In this study, the adhesion index was assessed by comparing the initial bacterial counts to those adhering to Caco-2 cells after a 2 h incubation period. Polysaccharide supplementation significantly enhanced the adhesion of probiotics to gut epithelial cells when compared to the control, with the highest adhesion observed at a 2% polysaccharide concentration (Figure 16A). To evaluate the competitive inhibitory effects of polysaccharides against enteropathogenic bacteria, *E. coli* was co-incubated with probiotics on a Caco-2 monolayer. The results showed significant reductions in *E. coli* attachment when the probiotics were treated with polysaccharides, underscoring their ability to enhance probiotic competitiveness against pathogens (Figure 16B). Collectively, these findings demonstrate that DpMP and DbMP supplementation enhances probiotic adhesion to gut epithelial cells while protecting against pathogen attachment, such as *E. coli*. This dual action improves probiotic efficacy, supports gut epithelial adherence, and strengthens defenses against harmful pathogens, highlighting the potential of DpMP and DbMP in promoting gut health and preventing enteropathogen-induced infections.

## 4. Conclusions

This study highlights the anti-inflammatory and prebiotic properties of the Korean yam (*D. polystachya* and *D. bulbifera*). Among the various extraction methods, the 95% ethanol extracts exhibited superior anti-inflammatory properties by significantly suppressing NO production, ROS levels, iNOS expression, and the pro-inflammatory mediators in LPS-stimulated RAW 264.7 cells. These extracts effectively modulated the key inflammatory signaling pathways, including MAPK and NF-κB, while reducing JNK phosphorylation, thereby emphasizing their potent anti-inflammatory mechanisms. Polysaccharides were extracted from *D. polystachya* and *D. bulbifera* and subsequently characterized for their biological activities. The polysaccharides from both yam species were analyzed for their glucose and uronic acid content as well as monosaccharide composition. These polysaccharides significantly enhanced immune responses by stimulating macrophage activation, increasing ROS and NO production, and upregulating the expression of immune-related proteins such as iNOS, COX-2, IL-1β, and TNF-α. Additionally, they demonstrated prebiotic properties by promoting the growth of probiotics, enhancing probiotic adhesion to gut epithelial cells, and inhibiting the attachment of *E. coli*. These findings suggest that *D. polystachya* and *D. bulbifera* extracts and polysaccharides hold significant promise as candidates for developing functional foods and nutraceuticals aimed at managing inflammation, boosting immunity, and supporting gut health. Further studies are warranted to confirm their in vivo efficacy, which will be reported separately.

## Figures and Tables

**Table 1:** Extraction yields of various extracts from *D. polystachya* and *D. bulbifera*.

Sample	Ethanol		Water	
	50%	95%	75 °C	95 °C
*D. polystachya*	10.6%	2.1%	6.93%	6.2%
*D. bulbifera*	8.3%	1.3%	4.4%	3%

**Table 2:** Yield and chemicals properties of DpMP and DbMP.

	Yield (%)	Total Carbohydrates (%)	Total Protein (%)	Uronic Acid (%)
DpMP	0.93 ± 0.31	26.44 ± 1.21	20.83 ± 1.51	19.18 ± 0.93
DbMP	4.05 ± 0.96	22.16 ± 1.47	23.43 ± 1.50	13.48 ± 0.51

All the experiments were carried out in triplicate (*n* = 3) and the results are expressed as mean ± S.D.

**Table 3:** Monosaccharide composition of DpMP and DbMP.

Ratio (%)	Galacturonic Acid	Glucose	Galactose	Mannose
DpMP	69.98	8.84	18.38	2.80
DbMP	68.09	7.22	22.09	2.60

## Data Availability

The original contributions presented in the study are included in the article, further inquiries can be directed to the corresponding author.
